# Minimal extrathyroidal extension affects the prognosis of differentiated thyroid cancer: Is there a need for change in the AJCC classification system?

**DOI:** 10.1371/journal.pone.0218171

**Published:** 2019-06-14

**Authors:** Zeming Liu, Yihui Huang, Sichao Chen, Di Hu, Min Wang, Ling Zhou, Wei Zhou, Danyang Chen, Haifeng Feng, Wei Wei, Chao Zhang, Wen Zeng, Liang Guo

**Affiliations:** 1 Department of Plastic Surgery, Zhongnan Hospital of Wuhan University, Wuhan, China; 2 Department of Pediatrics, St John Hospital and Medical Center, Detroit, MI, United States of America; 3 Department of Cardiovascular Surgery, Union Hospital, Tongji Medical College, Huazhong University of Science and Technology, Wuhan, China; 4 Department of Ophthalmology, Zhongnan Hospital of Wuhan University, Wuhan, China; University of Cincinnati College of Medicine, UNITED STATES

## Abstract

Minimal extrathyroidal extension (ETE) is defined as tumor cells extending to the sternothyroid muscle or perithyroidal soft tissue. However, there is controversy regarding whether the magnitude of ETE (minimal or gross) should be considered in assigning a precise TNM stage to patients with thyroid cancer in the seventh/eighth editions of the AJCC system. The present study evaluated Surveillance, Epidemiology, and End Results data from 107,114 patients with differentiated thyroid cancer (2004–2013) to determine whether the magnitude of ETE (thyroid confinement, minimal, or gross) influenced the ability to predict cancer-specific survival (CSS) and overall survival (OS). Patient mortality was evaluated using Cox proportional hazards regression analyses and Kaplan-Meier analyses with log-rank tests. The cancer-specific mortality rates per 1,000 person-years were 1.407 for the thyroid confinement group (95% CI: 1.288–1.536), 5.133 for the minimal ETE group (95% CI: 4.301–6.124), and 29.735 for the gross ETE group (95% CI: 28.147–31.412). Relative to the thyroid confinement group, patients with minimal ETE and gross ETE had significantly poorer CSS and OS in the univariate and multivariate analyses (both P<0.001). After propensity-score matching according to age, sex, and race, we found that thyroid confinement was associated with better CSS and OS rates than minimal ETE (P<0.001) and gross ETE (P<0.001). These results from a population-based cohort provide a reference for precise personalized treatment and management of patients with minimal ETE. Furthermore, it may be prudent to revisit the magnitude of ETE as advocated by the AJCC and currently used for treatment recommendation by the American Thyroid Association.

## Introduction

The incidence of thyroid cancer has generally been increasing during recent decades, although the mortality rate has steadily declined [1]. For example, during 2008–2012, most countries had age-standardized mortality rates of 0.20–0.40/100,000 men and 0.20–0.60/100,000 women, with steady increases among both sexes in the incidence of thyroid cancer (mainly papillary carcinoma) [1]. Differentiated thyroid carcinomas include papillary and follicular thyroid carcinomas [2], which are among the most curable of all cancers. However, some patients have a high risk of recurrence or even death from papillary and follicular thyroid carcinomas [3], with specific clinicopathological features being associated with progression and a dire prognosis even after extensive surgery, radioactive iodine (RAI) ablation therapy, and thyroid-stimulating hormone suppression [4]. Thus, well-established clinicopathological indicators must be used to predict patient prognosis and select treatment for differentiated thyroid carcinoma.

Previous research has indicated that appropriate treatment for thyroid cancer should be selected based on the precise TNM stage [4–6]. In this context, minimal extrathyroidal extension (ETE) is defined as tumor cells extending to the sternothyroid muscle or perithyroidal soft tissue [7]. Furthermore, there is significant debate regarding whether ETE should be incorporated into the T status for differentiated thyroid cancer, especially as the definitions of thyroid cancer T status were changed in 2002 for the AJCC and UICC systems [8, 9]. The fifth edition of the UICC system defined T3 disease as a >4-cm tumor without any ETE and T4 disease as any tumor with ETE. In contrast, the sixth edition of the UICC system defined T3 disease as a tumor with a greatest dimension of >4 cm or any tumor with minimal ETE, while T4a disease was defined as tumors extending beyond the thyroid capsule and with invasion of the subcutaneous soft tissue, larynx, trachea, esophagus, or recurrent laryngeal nerve [10]. The eighth edition of the AJCC system was published in 2018 and defines T3 disease as including gross ETE, while the seventh edition defined T4 disease as including gross ETE [11–13]. Interestingly, while gross ETE plays an increasingly significant role in the AJCC system, minimal ETE was removed from the definition of T3 disease in the eighth edition [11]. Thus, there remains controversy regarding whether the magnitude of ETE (i.e., minimal vs. gross) should be considered in assigning a precise TNM stage to patients with thyroid cancer, and further studies are needed to determine the impact of ignoring minimal ETE and only considering gross ETE when assigning a T status. The present study evaluated Surveillance, Epidemiology, and End Results (SEER) data to determine whether the magnitude of ETE influenced the ability to predict cancer-specific survival (CSS) and overall survival (OS) among patients with differentiated thyroid cancer.

## Materials and methods

This study’s retrospective protocol was approved by Zhongnan Hospital and Union hospital’s ethical review board and complied with the ethical standards of the Declaration of Helsinki, as well as the relevant national and international guidelines. The study cohort was comprised of 107,114 patients with differentiated thyroid cancer from the SEER database (2004–2013). Among these patients, the extent of tumor extension was classified as confinement to the thyroid parenchyma (80.5%), minimal ETE (6.0%), or gross ETE (10.2%). A total of 3,529 patients were excluded based on missing or unknown data regarding tumor extension.

The patients’ demographic parameters were defined as sex (male and female), race (white, black, and other or unknown), and age at diagnosis (≤55 years or >55 years). Lymph node and distant metastases were classified as present or absent. Treatment characteristics were defined as surgery type (none, lobectomy, subtotal or near-total thyroidectomy, and total thyroidectomy), and radiation therapy (none, radiation beam or radioactive implants, and radioisotopes or radiation beam with isotopes or implants).

The patients’ follow-up data were evaluated to calculate the rates of CSS and OS, as well as the mortality rates per 1,000 person-years. Univariate and multivariate Cox regression analyses were performed to examine whether CSS and OS were associated with age, sex, race, histological type, TNM stage, extension status, radiation treatment, or surgery type. Survival curves were generated using the Kaplan-Meier method and analyzed using the log-rank test. To minimize selection bias, propensity-score matching was performed for all relevant factors. Among the whole cohort, the propensity scores were calculated using a multivariate probit regression model that included three sets of relevant risk factors. The first set included age, sex, and race. The second set included the factors from the first set plus TNM stage and multifocality. The third set included the factors from the second set plus surgery type and radiation treatment. Matching was performed using a 1:1 matching protocol with a caliper of 0.1 standard deviations for the probit values. Statistical analyses were performed using SPSS software (version 23.0), StataSE software, and GraphPad Prism software (version 6). Differences were considered statistically significant at P-values of <0.05.

## Results

### Demographic and clinicopathological characteristics

The patients’ mean ages and follow-up durations according to the magnitude of ETE are shown in Table 1. Patients with minimal ETE were clearly older than patients with gross ETE. However, there were no significantly differences in the histological types between patients with minimal ETE and gross ETE.

**Table 1:** Characteristics for patients with different extent of tumor extension.

Covariate	level	Extent of tumor extension							
		Minimal ETE(n = 6395)	%	Confinement(n = 86266)	%	P Value	Gross ETE(n = 10924)	%	P Value
Age at diagnosis		49.99±16.03		49.45±15.24		<0.001	54.18±17.65		<0.001
Year of diagnosis	2004–2008	2365	37	36173	41.9	<0.001	5386	49.3	<0.001
	2009–2013	4030	63	50093	58.1		5538	50.7	
Sex	Female	4621	72.3	66955	77.6	<0.001	7388	67.6	<0.001
	Male	1774	27.7	19311	22.4		3536	32.4	
Race	White	5132	81.2	70750	83.1	<0.001	8822	81.3	<0.001
	Black	242	3.8	6049	7.1		560	5.2	
	Other	948	15	8376	9.8		1466	13.5	
Histological Types	Papillary	5903	96.8	76381	93.7	<0.001	9014	96.3	0.068
	Follicular	193	3.2	5157	6.3		348	3.7	
T stage	T1	18	0.3	58892	70.2	<0.001	28	0.3	<0.001
	T2	4	0.1	17490	20.9		1	0	
	T3	6321	98.9	7223	8.6		6248	57.3	
	T4	48	0.8	266	0.3		4625	42.4	
N-Stage	N0	3179	50.3	71590	84.3	<0.001	4576	43.4	<0.001
	N1	3135	49.7	13289	15.7		5971	56.6	
M-Stage	M0	6104	97.4	84418	99.2	<0.001	9495	89.4	<0.001
	M1	162	2.6	716	0.8		1131	10.6	
Multifocality	Yes	2914	47.1	53657	63.4	<0.001	5103	49.5	0.003
	No	3275	52.9	30978	36.6		5211	50.5	
Radiation	None or refused	1697	27.4	47001	55.7	<0.001	3133	29.3	<0.001
	Radiation Beam or Rdioactive implants	207	3.3	1355	1.6		1161	10.9	
	Radioisotopes or Radiation beam plus isotopes or implants	4281	69.2	36032	42.7		6381	59.8	
Surgery	Lobectomy	314	5	13852	16.6	<0.001	661	6.6	<0.001
	Subtotal or near-total thyroidectomy	142	2.3	3376	4.1		366	3.7	
	Total thyroidectomy	5815	92.7	66095	79.3		8957	89.7	
Survival months (month)		45.03±32.755		49.71±33.858		<0.001	47.21±35.073		<0.001
CSS	Alive	6267	98	85713	99.4	<0.001	9448	86.5	<0.001
	Death	128	2	553	0.6		1476	13.5	
OS	Alive	6065	94.8	82277	95.4	0.05	8627	79	<0.001
	Death	330	5.2	3989	4.6		2297	21	

Confinement:confinement to the thyroid parenchyma; Minimal ETE: minimal extrathyroidal extension;Gross ETE: gross extrathyroidal extension

### Cancer-specific and all-cause mortality rates

The cancer-specific and all-cause mortality rates were significantly different in the two-group and three-group comparisons (Table 2). During the follow-up period, cancer-specific deaths were detected for 535 patients in the thyroid confinement group, 128 patients in the minimal ETE group, and 1,476 patients in the gross ETE group. The cancer-specific mortality rates per 1,000 person-years were 1.407 for the confinement group (95% confidence interval [CI]: 1.288–1.536), 5.133 for the minimal ETE group (95% CI: 4.301–6.124), and 29.735 for the gross ETE group (95% CI: 28.147–31.412) (Tables 2 and 3). During the follow-up period, all-cause deaths were detected for 3,989 patients in the thyroid confinement group, 330 patients in the minimal ETE group, and 2,297 patients in the gross ETE group. The all-cause mortality rates per 1,000 person-years were 9.9 for the confinement group (95% CI: 9.612–10.269), 13.4 for the minimal ETE group (95% CI: 12.046–14.987), and 47.0 for the gross ETE group (95% CI: 44.950–49.052) (Tables 2 and 3).

**Table 2:** Cancer-specific mortality and all-cause mortality according to different extent of tumor extension.

Classification		Cancer-Specific Mortality						All-cause Mortality					
		Cancer-Specific Mortality	%	HR 95%	CI		P value	All-cause Mortality	%	HR 95%	CI		P value
Ⅰ	Confinement	553	0.6	ref			<0.001	3989	4.6	ref			<0.001
	Minimal ETE	128	2	3.285	2.71	3.982	<0.001	330	5.2	1.283	1.146	1.437	<0.001
	Gross ETE	1476	13.5	22.077	20.016	24.35	<0.001	2297	21	5.069	4.812	5.34	<0.001
Ⅱ	Confinement + Minimal ETE	681	0.7	ref				4319	4.6	ref			
	Gross ETE	1476	13.5	19.173	17.506	21	<0.001	2297	21	4.979	4.73	5.241	<0.001
Ⅲ	Confinement	553	0.6	ref				3989	4.6	ref			
	Minimal ETE + Gross ETE	1604	9.2	15.16	13.759	16.702	<0.001	2627	15	3.701	3.521	3.891	<0.001

Confinement:confinement to the thyroid parenchyma; Minimal ETE: minimal extrathyroidal extension;Gross ETE: gross extrathyroidal extension

**Table 3:** Hazard ratios of different extent of tumor extension for the cancer specific deaths and all cause deaths of differentiated thyroid cancer.

Extension	Cancer-Specific Mortality	%	Cancer-Specific Mortality	95%CI	All-cause Mortality	%	All-cause Mortality	95%CI
	No.		1,000 Person-Years		No.		1,000 Person-Years	
Confinement	553	0.60	1.407	1.288–1.536	3989	4.60	9.9	9.612–10.269
Minimal ETE	128	2.00	5.133	4.301–6.124	330	5.20	13.4	12.046–14.987
Gross ETE	1476	13.50	29.735	28.147–31.412	2297	21.00	47.0	44.950–49.052

Confinement:confinement to the thyroid parenchyma; Minimal ETE: minimal extrathyroidal extension;Gross ETE: gross extrathyroidal extension

### Clinicopathological parameters that affected CSS and OS

The univariate Cox regression analyses revealed that CSS was associated with age, sex, race, histological type, N-stage, M-stage, extension, multifocality, radiation treatment, and surgery type (all P<0.05, Table 4). Relative to the thyroid confinement group, patients with minimal ETE and gross ETE had significantly poorer CSS and OS in the univariate and multivariate analyses (all P<0.001, Tables 4 and 5). Race and surgery type were significantly associated with poorer CSS in the univariate analysis but not in the multivariate analysis (Table 4).

**Table 4:** Clinicopathologic parameters associated with the cancer-specific survival.

Parameters		HR	Univariate			HR	Multivariate		
			95% CI		p value		95% CI		p value
Year of diagnosis	2004–2008	ref				ref			
	2009–2013	0.801	0.738	0.87	<0.001	0.843	0.712	0.999	0.049
Age at diagnosis	< = 55	ref				ref			
	>55	10.026	9.023	11.14	<0.001	5.962	4.949	7.182	<0.001
Sex	Female	ref				ref			
	Male	2.393	2.215	2.586	<0.001	1.422	1.221	1.657	<0.001
Race	White	ref				ref			0.948
	Black	1.227	1.063	1.417	0.005	0.979	0.699	1.372	0.902
	Other	1.292	1.151	1.45	<0.001	1.032	0.833	1.278	0.773
Histological Types	Papillary	ref				ref			
	Follicular	2.903	2.486	3.39	<0.001	2.242	1.79	2.808	<0.001
N-Stage	N0	ref				ref			
	N1	4.906	4.503	5.346	<0.001	2.065	1.734	2.459	<0.001
M-Stage	M0	ref				ref			
	M1	50.921	46.893	55.294	<0.001	7.593	6.32	9.121	<0.001
Extension	Confinement	ref				ref			
	Minimal ETE	3.285	2.71	3.982	<0.001	3.709	2.784	4.941	<0.001
	Gross ETE	22.077	20.016	24.35	<0.001	9.318	7.658	11.338	<0.001
Multifocality	Yes	ref				ref			
	No	0.665	0.604	0.733	<0.001	0.743	0.637	0.865	<0.001
Radiation	None or refused	ref				ref			
	Radiation beam or radioactive implants	14.58	13.353	15.92	<0.001	3.435	2.725	4.33	<0.001
	Radioisotopes or radiation beam plus isotopes or implants	0.442	0.399	0.491	<0.001	0.804	0.671	0.964	0.018
Surgery	Lobectomy	ref				ref			0.143
	Subtotal or near total thyroidectomy	1.496	1.197	1.869	<0.001	1.281	0.869	1.889	0.211
	Total thyroidectomy	0.786	0.686	0.901	0.001	0.939	0.722	1.222	0.642

Confinement:confinement to the thyroid parenchyma; Minimal ETE: minimal extrathyroidal extension;Gross ETE: gross extrathyroidal extension

**Table 5:** Clinicopathologic parameters associated with the overall survival.

Parameters		HR	Univariate			HR	Multivariate		
			95% CI		p value		95% CI		p value
Year of diagnosis	2004–2008	ref				ref			
	2009–2013	0.878	0.833	0.926	<0.001	0.904	0.833	0.98	0.015
Age at diagnosis	< = 55	ref				ref			
	>55	8.176	7.713	8.668	<0.001	5.933	5.485	6.416	<0.001
Sex	Female	ref				ref			
	Male	2.363	2.256	2.476	<0.001	1.707	1.594	1.828	<0.001
Race	White	ref				ref			
	Black	1.328	1.223	1.442	<0.001	1.34	1.185	1.515	<0.001
	Other	0.979	0.906	1.058	0.6	0.821	0.73	0.924	0.001
Histological Types	Papillary	ref				ref			
	Follicular	1.794	1.64	1.961	<0.001	1.446	1.29	1.621	0.001
N-Stage	N0	ref				ref			
	N1	2.05	1.947	2.158	<0.001	1.416	1.298	1.546	<0.001
M-Stage	M0	ref				ref			
	M1	19.553	18.446	20.725	<0.001	4.511	3.973	5.121	<0.001
Extension	Confinement	ref				ref			
	Minimal ETE	1.283	1.146	1.437	<0.001	1.271	1.102	1.466	0.001
	Gross ETE	5.069	4.812	5.34	<0.001	2.341	2.142	2.56	<0.001
Multifocality	Yes	ref				ref			
	No	0.763	0.723	0.805	<0.001	0.938	0.875	1.006	0.073
Radiation	None or refused	ref				ref			
	Radiation beam or radioactive implants	5.732	5.367	6.122	<0.001	1.688	1.458	1.954	<0.001
	Radioisotopes or radiation beam plus isotopes or implants	0.458	0.434	0.484	<0.001	0.656	0.609	0.707	<0.001
Surgery	Lobectomy	ref				ref			0.049
	Subtotal or near total thyroidectomy	1.092	0.963	1.238	0.172	1.006	0.854	1.184	0.947
	Total thyroidectomy	0.724	0.675	0.777	<0.001	0.902	0.822	0.99	0.03

Confinement:confinement to the thyroid parenchyma; Minimal ETE: minimal extrathyroidal extension;Gross ETE: gross extrathyroidal extension

The univariate Cox regression analyses revealed that OS was also associated with age, sex, race, histological type, N-stage, M-stage, multifocality, extension, and radiation treatment (all P<0.001, Table 5). The univariate and multivariate Cox regression models revealed that poor OS was independently predicted by follicular thyroid carcinoma (univariate hazard ratio [HR]: 1.794, 95% CI: 1.640–1.961, P<0.001; multivariate HR: 1.446, 95% CI: 1.290–1.621, P = 0.001; Table 5). Relative to lobectomy, subtotal or near-total thyroidectomy did not significantly influence OS (univariate HR: 1.092, 95% CI: 0.963–1.238, P = 0.172; multivariate HR: 1.006, 95% CI: 0.854–1.184, P = 0.947; Table 5).

### Adjusting for patient features using propensity-score matching

**Fig 1:**
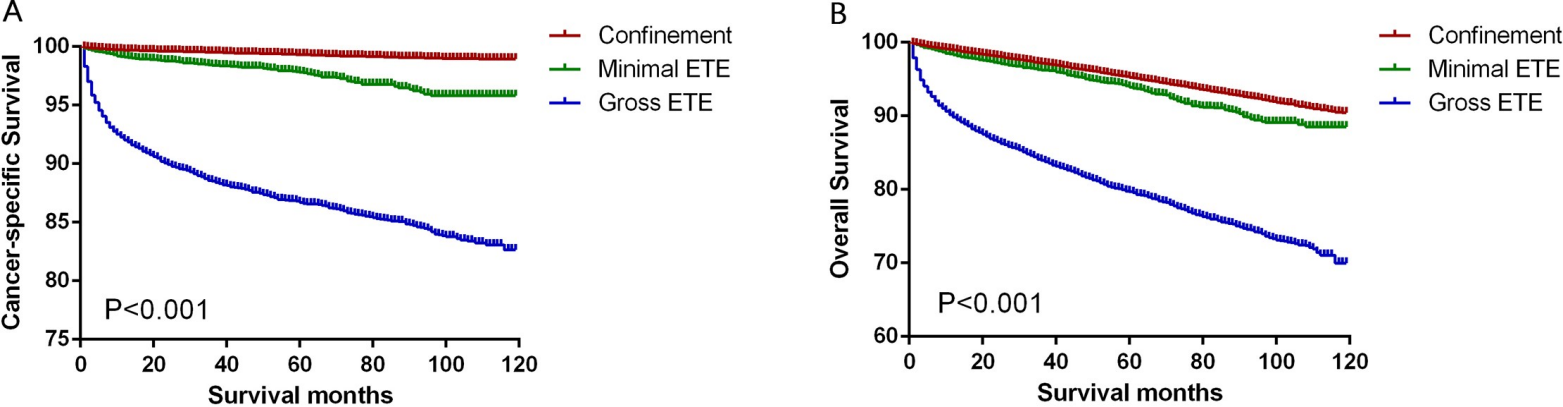
Kaplan-Meier curves for cancer-specific survival (A) and overall survival (B) among patients stratified according to subtype.

**Fig 2:**
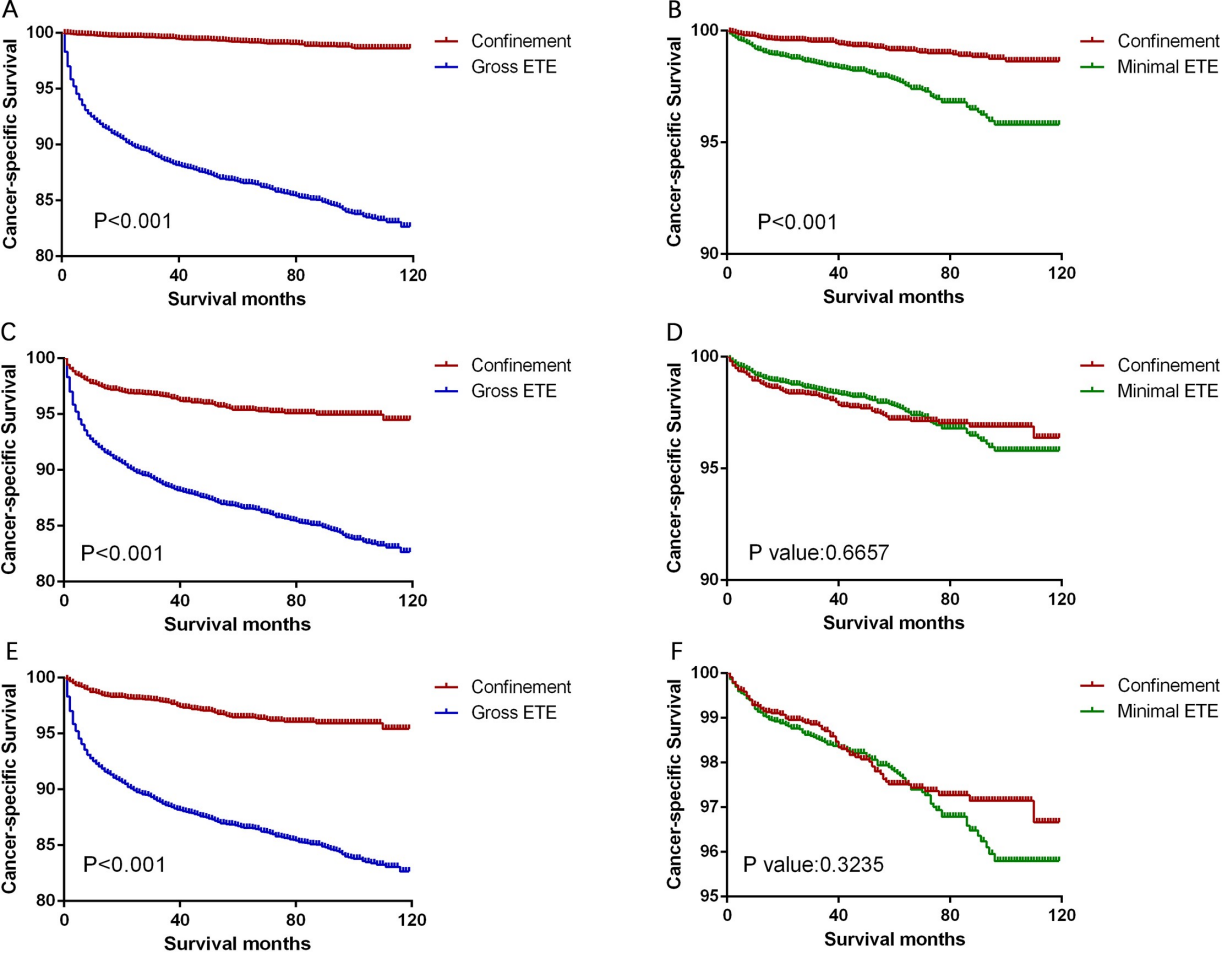
Kaplan-Meier curves for cancer-specific survival in the matched subtype pairs. Age, sex, and race matching was performed between the thyroid confinement and gross ETE groups (A) and between the thyroid confinement and minimal ETE groups (B). Age, sex, race, TNM stage, and multifocality matching was performed between the thyroid confinement and gross ETE groups (C) and between the thyroid confinement and minimal ETE groups (D). Age, sex, race, TNM stage, multifocality, surgery, and radiation treatment matching was performed between the thyroid confinement and gross ETE groups (E) and between the thyroid confinement and minimal ETE groups (F).

**Fig 3:**
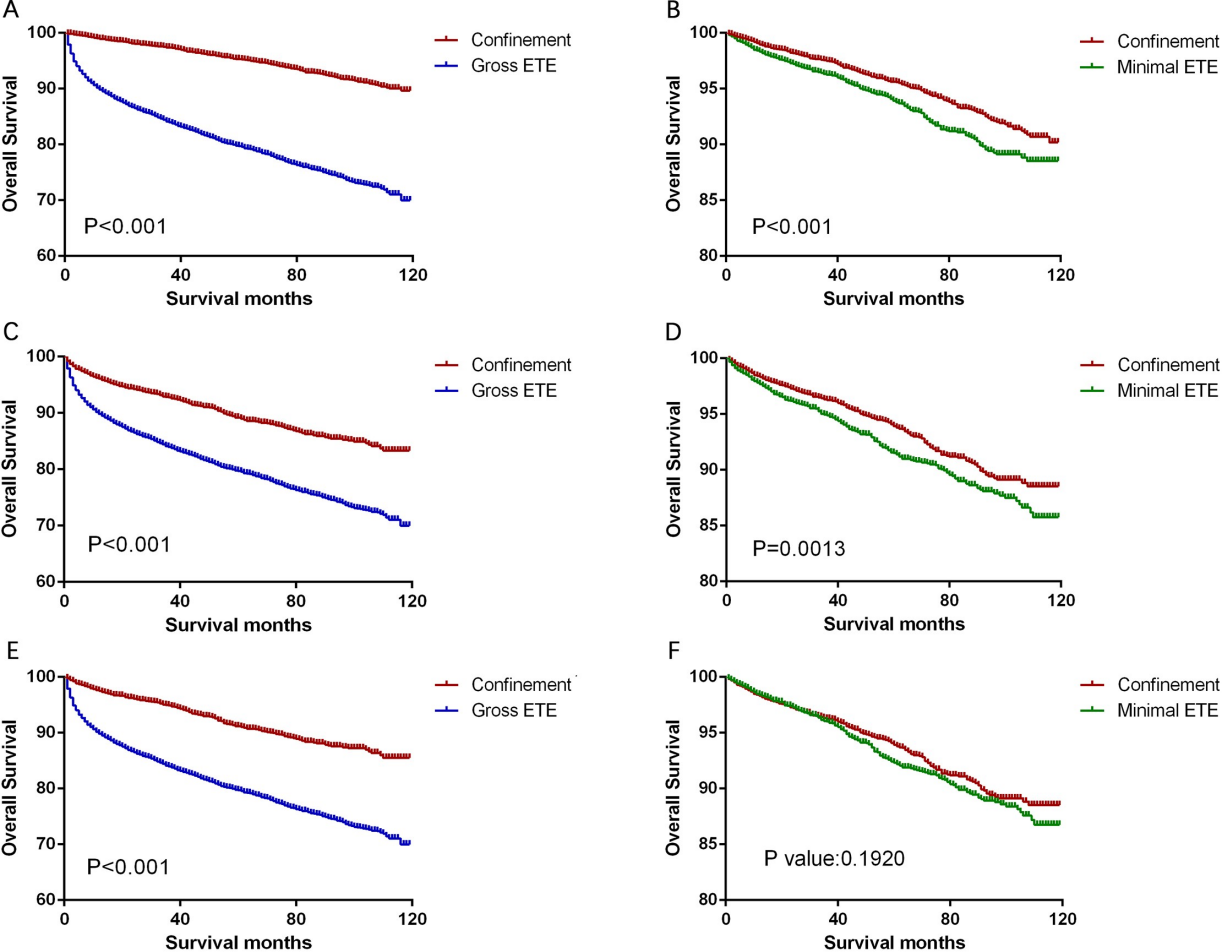
Kaplan-Meier curves for overall survival in the matched subtype pairs. Age, sex, and race matching was performed between the thyroid confinement and gross ETE groups (A) and between the thyroid confinement and minimal ETE groups (B). Age, sex, race, TNM stage, and multifocality matching was performed between the thyroid confinement and gross ETE groups (C) and between the thyroid confinement and minimal ETE groups (D). Age, sex, race, TNM stage, multifocality, surgery, and radiation treatment matching was performed between the thyroid confinement and gross ETE groups (E) and between the thyroid confinement and minimal ETE groups (F).

Significant differences in CSS and OS were observed when we compared the groups according to the magnitude of extension (all P<0.001, Fig 1A and 1B). Thus, to minimize selection bias, we performed propensity-score matching based on the three sets of covariates. After matching according to age, sex, and race (the first set of covariates), we found that thyroid confinement was associated with better CSS and OS rates than minimal ETE (P<0.001, Figs 2B and 3B) and gross ETE (P<0.001, Figs 2A and 3A). After matching according to age, sex, race, TNM stage, and multifocality (the second set of covariates), we found that gross ETE was associated with a lower CSS rate than thyroid confinement (P<0.001, Fig 2C) and a similar result was observed for OS (P<0.001, Fig 3C). Furthermore, similar results were obtained after matching for the third set of covariates, which included the previous factors plus radiation treatment and surgery type (all P<0.001, Figs 2E and 3E). Minimal ETE remained associated with a lower OS rate (P = 0.0013, Fig 3D), although there was no significant difference in the CSS rate between the minimal ETE and thyroid confinement groups (P = 0.666, Fig 2D). Moreover, after matching for all relevant factors, we failed to detect significant differences between the minimal ETE and thyroid confinement groups in terms of the CSS rate (P = 0.324, Fig 2F) and the OS rate (P = 0.192, Fig 3F). The negative results after matching for age, sex, race, TNM stage, and multifocality (Fig 2D and 2F), and after matching for all relevant factors (Fig 3F), indicate that these variables account for the decreased CSS and OS associated with minimal ETE.

## Discussion

Cases with ETE can be classified as minimal ETE (i.e., extension to the thyroid capsule, sternothyroid muscle, or perithyroidal soft tissue) or gross ETE (i.e., extension to the subcutaneous soft tissue, larynx, esophagus, trachea, recurrent laryngeal nerve, prevertebral fascia, mediastinal vessels, or carotid arteries) [14]. The magnitude of ETE has historically been thought to influence the assignment of T status, which in turn influences the TNM stage and selection of treatment options. However, it is difficult to define the precise boundary of extrathyroid and intrathyroidal tissues when determining the extent of cancer invasion, as both living patients and autopsy cases have failed to exhibit a complete or continuous fibrous capsule around the thyroid gland. This is a source of concern at our institution, as patients with AJCC stage III and stage IV tumors are routinely treated using RAI remnant ablation, or using external beam radiation therapy if they are older and have RAI-resistant tumors [15, 16]. Thus, there is debate regarding the importance of ETE in predicting the progression of differentiated thyroid carcinoma.

Recent research has indicated that ETE exerts only a minor effect on the rate of recurrence-free survival and related disease management decisions [8–10, 17]. Thus, minimal ETE was removed from the eighth edition of the AJCC system, with a greater emphasis placed on gross ETE. However, the present study’s univariate and multivariate analyses revealed that patients with minimal ETE had significantly lower rates of CSS and OS than patients with thyroid confinement. Moreover, patients with gross ETE had poorer rates of CSS and OS than patients with minimal ETE. However, the influence of minimal ETE on prognosis appears to be accounted for by other known variables including age, sex, race, TNM stage, and multifocality. Whether minimal ETE should be omitted from the AJCC system, as is the case in the eighth edition, deserves further study.

Woo et al. have reported that the presence or absence of minimal ETE did not significantly influence recurrence-free survival among patients with solitary papillary thyroid carcinoma or microcarcinoma [17]. However, that study had a small sample size and did not consider follicular thyroid cancer. More importantly, that study also failed to incorporate propensity-score matching in order to minimize the effect of potentially confounding factors. In contrast, the SEER database includes patient data from diverse geographical regions and is considered the gold-standard database for tumor surveillance and related analysis in the US, as it contains data from approximately 10% of the American patient population [18]. Therefore, the present study provides greater clarity regarding the prognostic implications of the magnitude of ETE among patients with differentiated thyroid cancer.

Several cohort studies have indicated that using a diagnostic age of 55 years as the prognostic cut-off provided better discrimination than using a cut-off of 45 years. Thus, patients are now classified into low- and high-risk groups based on ages of ≤55 years and >55 years [11]. The present study also used this cut-off value for the univariate and multivariate Cox regression analyses, and the results confirmed that an older age at diagnosis was associated with poorer CSS and OS rates.

Most studies have agreed that RAI ablation is effective for improving outcomes among patients with ETE to the sternothyroid muscle (i.e., minimal ETE), although few reports have examined the prognostic significance of ETE to the thyroid capsule [19–21]. Based on the outcomes of our study, we suggest that minimal ETE is associated with poorer CSS and OS outcomes. Thus, omitting minimal ETE from the definition of T3 disease would likely result in these patients being less likely to undergo RAI ablation, which appears problematic. Therefore, additional research is needed to refine the classification of ETE, to better understand the effects of minimal ETE, and to improve the personalized treatment of thyroid cancer.

The present study has several limitations. First, the SEER database lacks accurate information regarding the precise magnitude of ETE, and we could not perform specific analyses of the ETE subgroups. Second, we did not account for biochemical and genetic factors, recurrence, or surgery-related comorbidities in our analyses. Third, the SEER database does not include information regarding whether the patients underwent repeated surgery as well as detailed information concerning the radioactive iodine (RAI) therapy, and this lack of information may have biased our findings. Fourth, the propensity-score matching protocol and caliper size might have influenced the outcomes, and it is unclear whether the goodness-of-fit of the propensity score estimation model was sufficient.

## Conclusion

In conclusion, we suggest that minimal ETE may be associated with an increased risk of poor CSS and OS outcomes. In addition, patients with gross ETE have a greater risk of poor outcomes than patients with minimal ETE or thyroid confinement. Therefore, we believe that further discussion and research are needed to examine the omission of minimal ETE from the current AJCC staging system.

## References

[1] La Vecchia C, Malvezzi M, Bosetti C, Garavello W, Bertuccio P, Levi F, et al Thyroid cancer mortality and incidence: a global overview. Int J Cancer. 2015;136(9):2187–95. Epub 2014/10/07. 10.1002/ijc.29251 . 25284703

[2] Liu Z, Zeng W, Huang L, Wang Z, Wang M, Zhou L, et al Prognosis of FTC compared to PTC and FVPTC: findings based on SEER database using propensity score matching analysis. Am J Cancer Res. 2018;8(8):1440–8. Epub 2018/09/14. 30210915 PMC6129489

[3] Schlumberger MJ, Torlantano M. Papillary and follicular thyroid carcinoma. Baillieres Best Pract Res Clin Endocrinol Metab. 2000;14(4):601–13. Epub 2001/04/06. 10.1053/beem.2000.0105 . 11289737

[4] Ito Y, Ichihara K, Masuoka H, Fukushima M, Inoue H, Kihara M, et al Establishment of an intraoperative staging system (iStage) by improving UICC TNM classification system for papillary thyroid carcinoma. World J Surg. 2010;34(11):2570–80. Epub 2010/07/14. 10.1007/s00268-010-0710-2 . 20625728

[5] Mazzaferri EL. Managing small thyroid cancers. JAMA. 2006;295(18):2179–82. Epub 2006/05/11. 10.1001/jama.295.18.2179 . 16684990

[6] Pelizzo MR, Boschin IM, Toniato A, Piotto A, Bernante P, Pagetta C, et al Papillary thyroid microcarcinoma (PTMC): prognostic factors, management and outcome in 403 patients. Eur J Surg Oncol. 2006;32(10):1144–8. Epub 2006/07/29. 10.1016/j.ejso.2006.07.001 . 16872798

[7] Edge SB, Compton CC. The American Joint Committee on Cancer: the 7th edition of the AJCC cancer staging manual and the future of TNM. Ann Surg Oncol. 2010;17(6):1471–4. Epub 2010/02/25. 10.1245/s10434-010-0985-4 . 20180029

[8] Ito Y, Tomoda C, Uruno T, Takamura Y, Miya A, Kobayashi K, et al Minimal extrathyroid extension does not affect the relapse-free survival of patients with papillary thyroid carcinoma measuring 4 cm or less over the age of 45 years. Surg Today. 2006;36(1):12–8. Epub 2005/12/27. 10.1007/s00595-005-3090-8 . 16378187

[9] Hay ID, Johnson TR, Thompson GB, Sebo TJ, Reinalda MS. Minimal extrathyroid extension in papillary thyroid carcinoma does not result in increased rates of either cause-specific mortality or postoperative tumor recurrence. Surgery. 2016;159(1):11–9. Epub 2015/10/31. 10.1016/j.surg.2015.05.046 . 26514317

[10] Dobert N, Menzel C, Oeschger S, Grunwald F. Differentiated thyroid carcinoma: the new UICC 6th edition TNM classification system in a retrospective analysis of 169 patients. Thyroid. 2004;14(1):65–70. Epub 2004/03/11. 10.1089/105072504322783867 . 15009916

[11] Shah JP, Montero PH. New ajcc/uicc staging system for head and neck, and thyroid cancer. Revista Médica Clínica Las Condes. 2018;29(4):397–404. 10.1016/j.rmclc.2018.07.002

[12] Lydiatt WM, Patel SG, O'Sullivan B, Brandwein MS, Ridge JA, Migliacci JC, et al Head and Neck cancers-major changes in the American Joint Committee on cancer eighth edition cancer staging manual. CA Cancer J Clin. 2017;67(2):122–37. Epub 2017/01/28. 10.3322/caac.21389 . 28128848

[13] Doescher J, Veit JA, Hoffmann TK. [The 8th edition of the AJCC Cancer Staging Manual: Updates in otorhinolaryngology, head and neck surgery]. HNO. 2017;65(12):956–61. Epub 2017/07/19. 10.1007/s00106-017-0391-3 . 28717958

[14] Shin JH, Ha TK, Park HK, Ahn MS, Kim KH, Bae KB, et al Implication of minimal extrathyroidal extension as a prognostic factor in papillary thyroid carcinoma. Int J Surg. 2013;11(9):944–7. Epub 2013/07/04. 10.1016/j.ijsu.2013.06.015 . 23820062

[15] Jeon HM, Lim BJ, Chang HS, Hong S. The definition of minimal extrathyroid extension in thyroid pathology by analyzing sizable intra- and extrathyroid blood vessels. Korean J Pathol. 2012;46(6):548–53. Epub 2013/01/17. 10.4132/KoreanJPathol.2012.46.6.548 23323105 PMC3540332

[16] Radowsky JS, Howard RS, Burch HB, Stojadinovic A. Thyroid. 2014;24(2):241–4. Epub 2013/05/30. 10.1089/thy.2012.0567 . 23713855

[17] Woo CG, Sung CO, Choi YM, Kim WG, Kim TY, Shong YK, et al Clinicopathological Significance of Minimal Extrathyroid Extension in Solitary Papillary Thyroid Carcinomas. Ann Surg Oncol. 2015;22 Suppl 3:S728–33. Epub 2015/06/17. 10.1245/s10434-015-4659-0 26077913 PMC4686556

[18] Xiong Y, Zhao Q, Li Z, Wang S, Guo H, Liu Z, et al Propensity score matching analysis of the prognosis for the rare oxyphilic subtype of thyroid cancer (Hurthle cell carcinoma). Oncotarget. 2017;8(60):101362–71. Epub 2017/12/20. 10.18632/oncotarget.20732 29254170 PMC5731880

[19] Jeon YW, Ahn YE, Chung WS, Choi HJ, Suh YJ. Radioactive iodine treatment for node negative papillary thyroid cancer with capsular invasion only: Results of a large retrospective study. Asia Pac J Clin Oncol. 2016;12(1):e167–73. Epub 2013/12/03. 10.1111/ajco.12159 . 24289279

[20] Rivera M, Ricarte-Filho J, Tuttle RM, Ganly I, Shaha A, Knauf J, et al Molecular, morphologic, and outcome analysis of thyroid carcinomas according to degree of extrathyroid extension. Thyroid. 2010;20(10):1085–93. Epub 2010/09/24. 10.1089/thy.2010.0174 20860430 PMC4984786

[21] Hu A, Clark J, Payne RJ, Eski S, Walfish PG, Freeman JL. Extrathyroidal extension in well-differentiated thyroid cancer: macroscopic vs microscopic as a predictor of outcome. Arch Otolaryngol Head Neck Surg. 2007;133(7):644–9. Epub 2007/07/20. 10.1001/archotol.133.7.644 . 17638775

